# Gold nanoparticles as efficient antimicrobial agents for *Escherichia coli* and *Salmonella typhi*

**DOI:** 10.1186/1752-153X-7-11

**Published:** 2013-01-19

**Authors:** Enrique Lima, Roberto Guerra, Víctor Lara, Ariel Guzmán

**Affiliations:** 1 Instituto de Investigaciones en Materiales, UNAM, Circuito exterior s/n, Cd. Universitaria, Del. Coyoacán, C.P. 04510, Distrito Federal, Mexico; 2 Universidad Autónoma Metropolitana, Iztapalapa, Av. San Rafael Atlixco No. 186, Col. Vicentina, CP 09340, México DF, Mexico; 3 Instituto Politécnico Nacional, ESIQIE, Av. IPN UPALM Edif. 7, Zacatenco, 07738, México D.F, Mexico

**Keywords:** Gold, Zeolite, Microbial, Porous materials

## Abstract

**Background:**

It is imperative to eliminate bacteria present in water in order to avoid problems in healthy. *Escherichia coli* and *Salmonella typhi* bacteria are two common pollutants and they are developing resistance to some of the most used bactericide. Therefore new biocide materials are being tested. Thus, gold nanoparticles are proposed to inhibit the growth of these two microorganisms.

**Results:**

Gold nanoparticles were supported onto clinoptilolite, mordenite and faujasite zeolites. Content of gold in materials varied between 2.3 and 2.8 wt%. The size, dispersion and roughness of gold nanoparticles were highly dependent of the zeolite support. The faujasite support was the support where the 5 nm nanoparticles were highly dispersed. The efficiency of gold-zeolites as bactericides of *Escherichia coli* and *Salmonella typhi* was determined by the zeolite support.

**Conclusions:**

Gold nanoparticles dispersed on zeolites eliminate *Escherichia coli* and *Salmonella typhi* at short times. The biocidal properties of gold nanoparticles are influenced by the type of support which, indeed, drives key parameters as the size and roughness of nanoparticles. The more actives materials were pointed out Au-faujasite. These materials contained particles sized 5 nm at surface and eliminate 90–95% of *Escherichia coli* and *Salmonella typhi* colonies.

## Background

There is an increasing interest in materials holding antimicrobial properties because in several fields the use of these materials is mandatory, e.g. in medicine [1-3]. The most common antimicrobial compounds are benzalkonium chloride, triclosan and silver [4-6] although other heavy metals are also used. Some antimicrobials incorporated to other materials have been applied as adhesives [7], window cleaners [8], textiles, and wallpaper gloves [9], among others.

The silver as antimicrobial has been used mainly as ion Ag + and also different delivery systems that release silver ions in a variety of concentrations have been explored [10,11]. Furthermore, the efficiency of metallic Ag particles to inhibit the growth of bacteria has been also reported. These particles are proposed as an alternative to materials with a silver ion release system because of the large variables that determine the release of ions and also because ions are reactive in several media, for instance they are solvated or coordinated to other ions easily. Thus, metallic silver has been incorporated to some supports and were reported as efficient biocide materials [12,13]. Other reason to incorporate metallic silver particles onto supports is to obtain high specific surface area and a high fraction of surface atoms of silver nanoparticles will lead to high bactericide activity when compared to bulk silver metal [14,15].

Unfortunately, in a parallel way, whereas scientists develop new efficient antimicrobial materials, there is no doubt that bacterial resistance to silver also is developed. Thus, other heavy metals, mainly copper, have been tested to kill bacteria. In this context, gold has been few explored as antimicrobial but it has been largely used as catalyst in last year’s [16,17]. The success of gold as catalyst is a consequence of the manipulation of this metal at the nanometric size, mainly stabilizing nanoparticles in different inorganic supports such as silica, alumina, zeolites. Use of gold in the killing of bacteria has been focused to some treatments of arthritis [18,19]. Medical applications of gold include the use of sulphur-gold compounds as anti-inflammatory [20,21]. It has been proposed that gold inhibit the proliferation of T cells by modifying the permeability of mitochondrial membrane [22,23]. Another proposal suggests that gold compounds limit the enzymatic activity of liposome in macrophages [24].

The gold, in a similar way that silver, when used in reasonable amounts, does not negatively affect the human body [20]. Therefore, we have started this work with the goal to explore the gold-supported antimicrobial activity for *Escherichia coli* and *Salmonella typhi*, which are two bacteria currently present in foods and water, being both them that have more and more resistance to silver-based antimicrobials. Here we report the results when gold was supported in faujasite, mordenite and clinoptilolite zeolites. We have selected these zeolites because they are easily available and differ regarding their physicochemical properties [25,26]. Thus, these selection leads to prepare a wide series of gold-supported materials and disclose on the most suitable conditions to find the most active antimicrobial material.

## Results and discussion

### Physicochemical properties of materials

**Figure 1:**
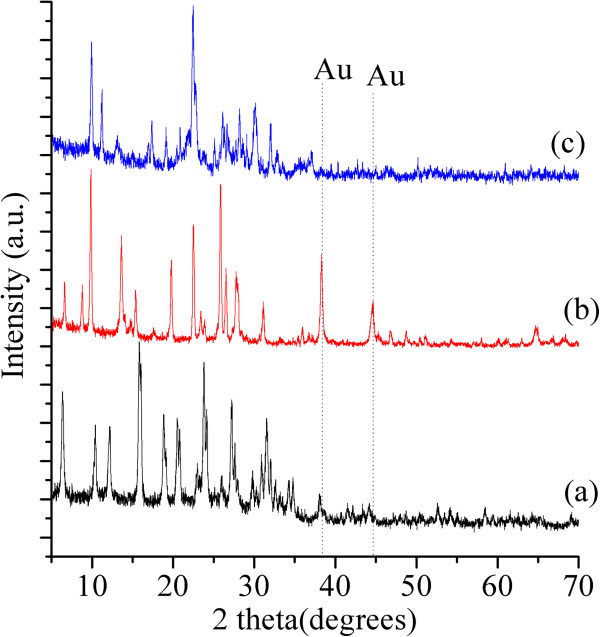
X-ray diffraction patterns of Au-zeolites (a) Au-Y (b) Au-M and (c) Au-C.

**Figure 2:**
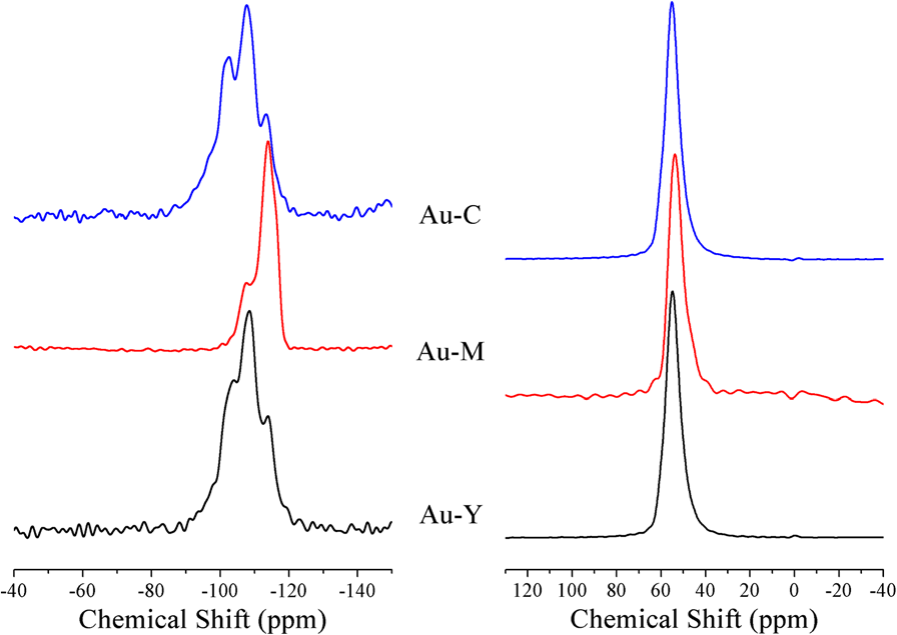
**On the left, **^**29**^**Si MAS NMR spectra and on the right the **^**27**^**Al MAS NMR spectra acquired at 79.46 and 104 MHz, respectively.**

Data in Table 1 shows that three Au-zeolite samples were not significantly different regarding the gold content. The greatest difference observed was 0.5 wt %, between gold clinoptilolite and mordenite materials. XRD patterns displayed in Figure 1 suggest that cristallinity differed for the Au-zeolite set. The sample Au-Y seems to be the sample with lowest crystallinity. Furthermore, only the XRD pattern of Au-M exhibited the peaks due to metallic gold (peaks labeled Au) meaning that on mordenite the gold formed big particles dispersed on the external surface and in other two samples the gold was better dispersed. Both, cristallinity and gold dispersion evidenced by XRD results are better understood if NMR and TEM results, respectively, are taken into account. For instance, in ^29^Si NMR spectra of Au-C sample (Figure 2) four resonance lines at −96, −102, −108.3 and −113.6 ppm were identified, which correspond to Si(3Al), Si(2Al), Si(1Al) and Si(0Al) configurations, respectively [27]. The relative intensities of these resonance peaks support that aluminum is well distributed in this sample and, because of the absence of a peak at chemical shifts as high as −90 ppm (due to Si(4Al)) units), no micro vicinities enriched in aluminum were present. Actually, this is the case of three Au-zeolites considered in this work. However, some differences should be remarked: the Au-M sample contains considerably less aluminum incorporated to zeolite framework. Note that in this sample each of silicon atoms is mainly connected to other silicon tetrahedral (resonance at −113.9 ppm) and few silicon atoms are connected to aluminum tetrahedral (peak at −107.29). Lastly, the sample Au-Y is similarly built as Au-C where the units Si(2Al) and Si(1Al) are the most abundant.

**Table 1:** Characteristics of Au zeolite samples under study

**Code sample**	**Type of support**	**Au gold content (wt %)**	**Specific surface area, BET (m**^**2**^**/g)**	**Fractal dimension of gold particles**
Au-Y	Faujasite Y	2.5	319	2.2
Au-M	Mordenite	2.8	177	2.8
Au-C	Clinoptilolite	2.3	88	2.4

^27^Al MAS NMR spectra, Figure 2 on the right, show that total of aluminum atoms are 4-fold coordinated to four oxygen atoms, peak at 55 ppm. Three Au-zeolite samples presented only the peak around 55 ppm in their ^27^Al MAS NMR spectra supporting that none of these three samples were dealuminated as a consequence of the gold loading. Furthermore, for three samples the width of the resonance peaks were similar suggesting that the amount of gold deposited close to aluminum does not differ significantly in the three samples.

**Figure 3:**
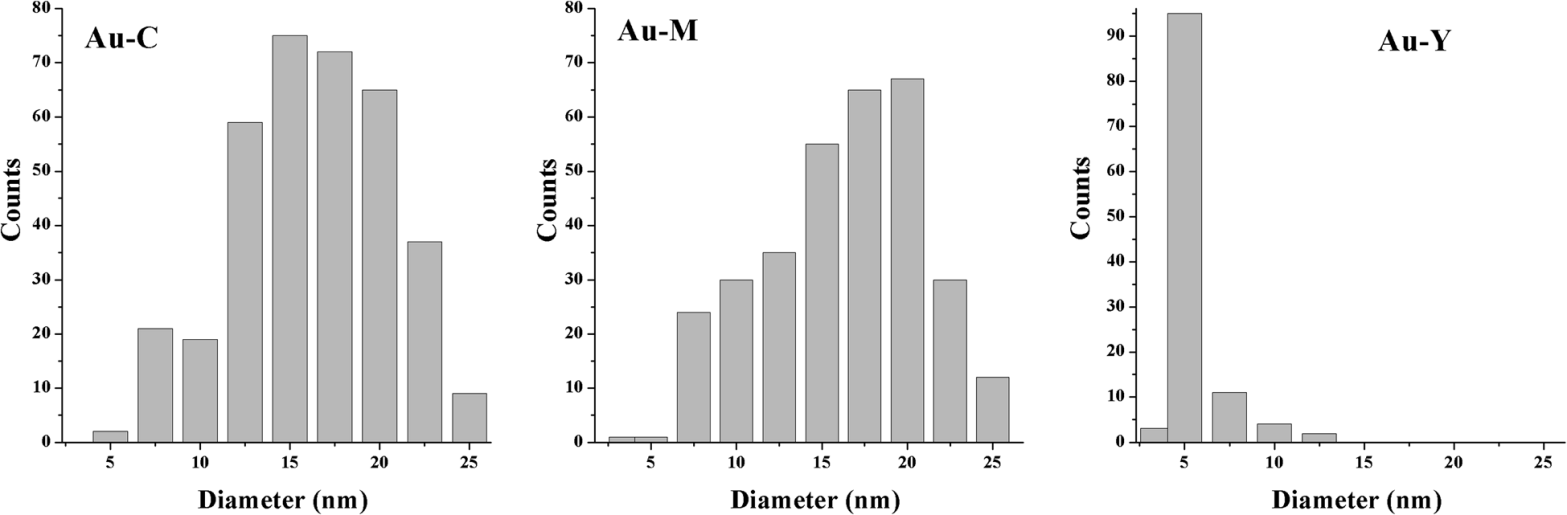
Diameter distribution of Au particles in Au-zeolite samples, as determined by TEM analysis.

The zeolite support determines the distribution size of gold particle (Figure 3) as obtained from TEM data. Au-C and Au-M materials have stabilized gold particles as small as 5–7.5 nm but the size particle distribution is very heterogeneous and the majority of particles are sized in the 10–20 nm range. In contrast, in the Au-Y sample the gold particles are more homogeneously distributed and the majority of particles measure 5 nm. It is not surprising that the particle size of gold particles is determined by support because of the geometry of zeolitic channels.

Regarding the textural properties, once again the materials differed as reported in Table 1. On the one hand, the Au-Y material is the one with the highest specific surface area (SSA), which agrees with the feature that the gold was well dispersed as evidenced by TEM. The Au-C and Au-M have significantly lower SSA than Au-Y confirming that the big gold particles formed in these samples blocked some of the micropores of zeolites. On the other hand, the fractal dimension values of gold particles also differed as a function of support. The Au-Y material seems to have the most suitable particles because their lowest fractal dimension (2.2) means that the surface/volume ratio is the highest, and then the metallic surface could be easier accessible than in the other materials [28,29]. Furthermore, the unusual high fractal dimension for particles in Au-M sample reveals that the particles are more compact and their surface is more roughed than in other samples. The roughness should be not a determinant parameter for the performance as bactericide but the compactness yes.

### Bactericide properties of materials

**Figure 4:**
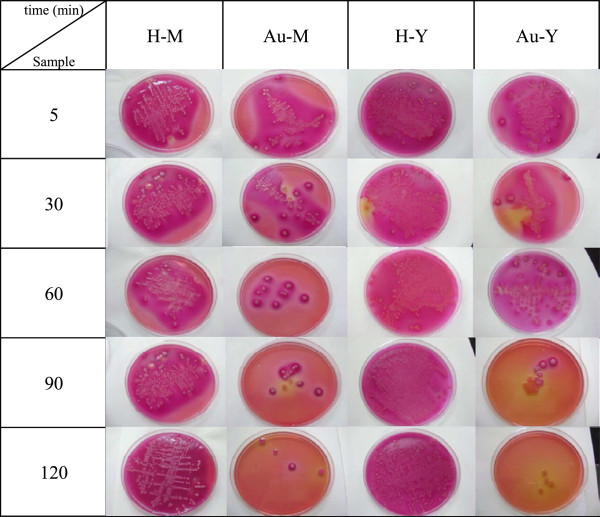
**Colonies formed by cell of *Escherichia coli *surviving after culture media was exposed to H-zeolites and Au-zeolites.**

**Figure 5:**
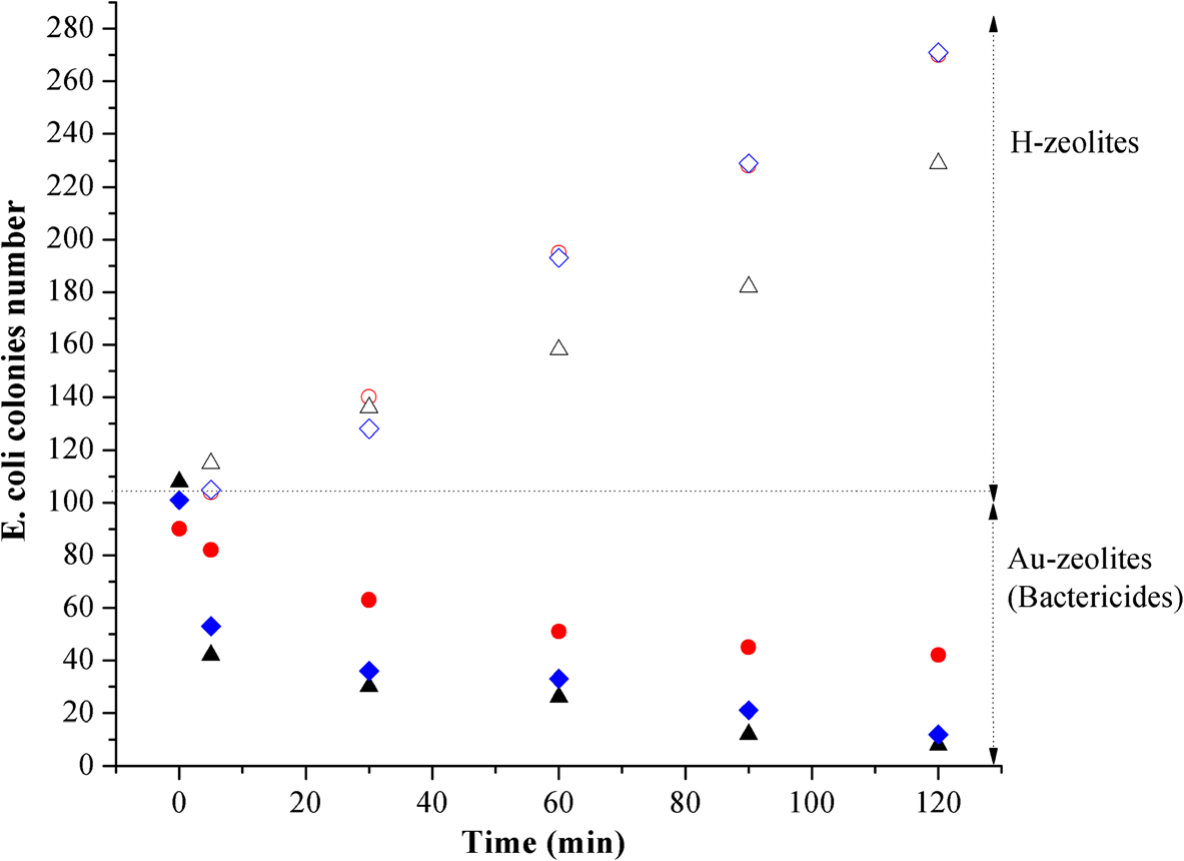
**Evolution as time went on of number of *Escherichia coli *colonies formed by cells survived in culture media in the presence of H-C (−o-), Au-C (−●-), H-M (−∆–), Au-M (−▲–), H-Y (−◊-) and Au-Y(−♦-).**

Figure 4 shows that the cells surviving formed colonies as time went on in the culture media in the presence of Au-zeolite samples and that the number of these colonies was a function of the zeolite support. Additionally, in Figure 5 are plotted the trend of elimination of bacteria as a function of time. Each point in the graph is the average value of five experiments carried out, non bar errors were included in the graphs because the greatest standard deviation was close to 3% and they are imperceptibles in the scale of the graph. From these figures, firstly, it has to be mentioned that none of the supports is intrinsically biocide materials as they all propitiates the growth of the bacteria, in this sense it is surprising that after two hours the initiated the experiment, on the gold-free zeolites, H-Y and H-C, the colonies number has been practically duplicated. Thus, the gold is the responsible of bactericide effect observed in all Au-zeolites, as shown by de decrease in the colonies number after times as short as 5 minutes. The performance as bactericide is determined by the type of support. Actually, the Au-M and Au-Y seem to be the best biocide materials as they allowed eliminate almost 90% of the colonies after 120 min. However, it should be mentioned that the Y support allows the highest growth of the bacteria, then it is clear that Au-Y is a better bactericide than the Au-M material, revealing that the dispersion and the roughness of the gold nanoparticles onto support lead the bactericide properties of the material. Comparing clinoptilolite and faujasite, Au-Y is better bactericide than Au-C, pointing out the importance of the dispersion of gold because both samples have smoothed particles but those on Au-Y are smaller, well dispersed on the support. Now, if the couple mordenite clinoptilolite are compared, mordenite is more efficient to kill *Escherichia coli* colonies than clinoptilolite. Au-M and Au-C contain gold nanoparticles with a similar size, but they differ regarding their fractal dimension, revealing that roughness is an important parameter driving the bactericide properties.

**Figure 6:**
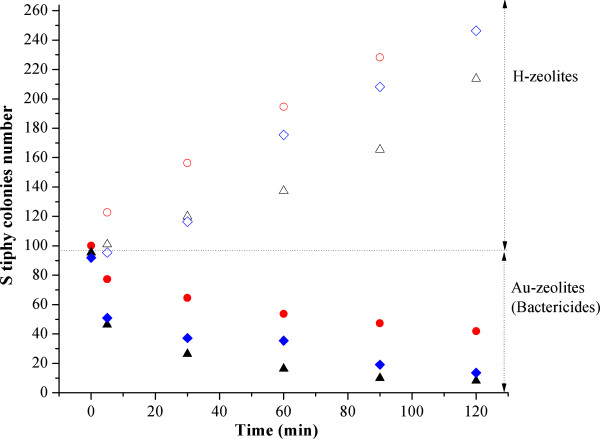
**Evolution as time went on of number of *Salmonella typhi *colonies formed by cells survived in culture media in the presence of H-C (−o-), Au-C (−●-), H-M (−∆–), Au-M (−▲–), H-Y (−◊-) and Au-Y (−♦-).**

Figure 6 displays the plots of number of colonies of *Salmonella typhi* formed by cells that survived after exposition to zeolitic materials for different times. In general, the Au-zeolites have also bactericide effects on *Salmonella typhi*, as observed for *Escherichia coli*. Indeed, samples have practically the same trend i.e. that once again the dispersion and roughness of gold nanoparticles are determinant to eliminate *Salmonella typhi*. The elimination’s rate, however, is slightly slower in *Salmonella typhi* than in *Escherichia coli*. This feature should be attributed to nature of bacteria because *Salmonella typhi* have a very resistant plasmatic membrane where the composition is very complex, roughly three proteins are implicated [30]. Actually, the most probably bactericide mechanism could be initiated by a variation of the electric field in the plasmatic membrane, which is totally possible because the Au particles are stabilized on protoned Y support, therefore the particles are not totally metallic, they are electron-deficient (δ^+^) as commonly occur when nanoparticles are supported in alumina and zeolites [31,32].

### Experimental

#### Materials

A clinoptilolite-rich tuff from Etla, Oaxaca in southeast Mexico was ground and sieved (0.15 mm). The zeolite was homogeneized in the protoned form (sample H-M). Both NH_4_^+^-Mordenite (trade name CBV 10A) and NH_4_^+^-Y faujasite (trade name CBV 300) zeolites with a SiO_2_/Al_2_O_3_ molar ratio of 13 and 5, respectively, were purchased from Zeolyst International (USA) and careful heating at 673 K to obtain protonated zeolites (H-M and H-Y).

The H-zeolite supports were suspended in a gold colloid solution (5 nm) purchased from Aldrich (USA). The suspension was stirred for 90 min, after that solid was separated by centrifugation, washed with distilled water, dried at 50°C and then calcined for 4 h and reduced at 500°C under a hydrogen flow. The final amount of Au in the Au-zeolites was around 2.5 wt %, similar amount was loaded in all samples, as determined by Inductively Coupled Plasma (ICP) analysis, Table 1. The code of Au loaded samples includes the prefix Au plus the code of the zeolite support, Table 1. These zeolites were used as bactericides following the procedure below described. In order to have control experiments, the zeolites without gold (H-zeolites) were also tested as bactericide materials, but, because the Au-zeolites preparation includes a step with a high thermal treatment, the H-zeolite were also thermal treated at 500°C keeping in mind the effect of the redistribution of extra-framework cations that currently occurs as a consequence of the temperature raising.

#### Characterization

Au-zeolites were characterized by X-ray diffraction (XRD), ^27^Al and ^29^Si solid-state nuclear magnetic resonance (NMR) under magic angle spinning (MAS) conditions, transmission electronic microscopy (TEM), nitrogen adsorption-desorption and small angle X-ray scattering (SAXS).

XRD patterns were obtained with a Bruker AXS D8 advance diffractometer coupled to a copper anode X-ray tube.

Solid-state ^27^Al MAS NMR single excitation spectra were acquired on a Bruker Avance 400 spectrometer at a frequency of 104.2 MHz. Short single pulses (π/12) were used. The samples were spun at 10 kHz, and the chemical shifts were referenced to an aqueous 1 M AlCl_3_ solution. ^29^Si MAS NMR spectra were acquired at 79.46 MHz using proton dipolar decoupling (HPDEC). Direct-pulsed NMR excitation was used throughout the experiment, employing 90° pulses (3 μs) with a pulse repetition time of 60 s. The spinning rate was 5 kHz, and the chemical shifts were referenced to tetra methyl silane.

Materials were analyzed by transmission electron microscopy in a 120 kV LEO-912AB (ZIES). The TEM images were processed digitally from the negative films by using a film scanner. Size distribution measurements for Au particles were performed on digital images by using the image analyzing software Image-Pro.

The specific surface areas were calculated by the Brunauer–Emmett–Teller (BET) method from nitrogen adsorption–desorption isotherms, measured at −196°C with an ASAP 2010 apparatus.

Small angle X-ray scattering experiments were performed using a Kratky camera coupled to a copper anode X-ray tube whose Kα radiation was selected with a nickel filter. The SAXS intensity data, I(h), were collected with a linear proportional counter. Then, they were processed with the ITP program [33-35] where the angular parameter, h, is defined as h = 4π sin θ/λ; θ and λ are the scattering angle and the X-ray wavelength, respectively. The fractal dimension of the scattering objects was evaluated from the slope of the curve logI(h) vs log(h).

The small-angle X-ray scattering may be due, as noticed by the Babinet principle, either too dense particles in a low-density environment or to pores or low-density inclusions in a continuous high electron density medium. Then, in order to characterize only the gold phase, we have subtracted the SAXS data of the free-gold zeolite from those of the gold-loaded zeolite. This method was earlier shown to be efficient in the characterization of particles supported onto porous materials [36-38].

#### Antimicrobial tests

*Escherichia coli* and *Salmonella typhi* were acquired from ENCB Mexico.

Tripticaseine broth medium was used for growing and maintaining the bacterial cultures. A starter culture of each strain was inoculated with fresh colonies and incubated for 24 h in Tripticaseine medium. The number of colonies formed by surviving cells was counted in a selective agar (MacConkey for *Escherichia coli* and brilliant green for *Salmonella typhi*). Fresh medium was inoculated in test tubes with the starter culture and grown at 35.5°C with continuous agitation at 30 rpm. The colonies were added to the tubes each 24 h in order to reach a control experiment where the classical exponential growth was observed as a function of time. Then, Au- zeolite was added to the culture, and samples of colonies were measured over a time course. Measurement proceeded as follows: The sample was seeded in Petri dishes previously loaded with 30 ml of selective agar. As a control, a culture plate was inoculated without bactericide material. The plates were incubated at 35.5°C under aerobic aerobic conditions and the colonies were counted. During all experiments with bacteria the material used was sterilized.

## Conclusion

Gold nanoparticles dispersed on zeolites are excellent biocide to eliminate *Escherichia coli* and *Salmonella typhi* at short times. The roughness and the dispersion of Au nanoparticles on the support are crucial parameters affecting the biocidal properties. The type of support is another important parameter in the effectiveness of the material to inhibit microorganisms. The more actives materials were pointed out Au-Y. These materials contained very small particles at surface actives to eliminate 90–95% of *Escherichia coli* and *Salmonella typhi* colonies at times as short as 90 minutes.

## Competing interests

The authors declare that they have no competing interests.

## Authors’ contributions

AG prepared the materials, measurements and data characterization. VL assisted during SAXS measurements and data evaluation of XRD measurements. RG carried out the experiments with bacteria. EL headed the scientific planning and evaluation of the project. All authors have read and approved the final version.
